# Assessing and addressing ongoing information and support needs among individuals with Lynch syndrome

**DOI:** 10.1186/1897-4287-9-S1-P20

**Published:** 2011-03-10

**Authors:** Anna M Leininger, Mia Resner, Carrie A Zabel

**Affiliations:** 1 HealthEast Cancer Care, Cancer Genetics Clinic, St. John’s Hospital, Maplewood, MN, USA; 2 William C Bernstein, MD Familial Cancer Registry, University of Minnesota, Minneapolis, MN, USA; 3 Mayo Clinic, Rochester, MN, USA

## Background

In 2008, Lynch syndrome patients requested referrals to a support organization, prompting the authors to assess available resources. While general information about Lynch syndrome was readily available from multiple sources including the internet, updated, detailed information and psychosocial support was largely unavailable. Based on patient responses to a subsequent needs assessment, an annual patient-oriented conference was organized. We report the results of the needs assessment and response to the conferences.

## Materials and methods

Five individuals with a diagnosis or family history of Lynch syndrome responded to a series of 20 questions intended to assess information and support needs as well as interest in attending a group meeting specific to Lynch syndrome. One participant provided a written response and four provided answers via telephone conversation. Responses were transcribed and reviewed for common elements.

## Results

Responders indicated interest in gathering with other people for the purpose of receiving updated information and support. Patient-identified areas of interest formed the basis of two (2009, 2010) 1-day, patient/family oriented conferences specific to Lynch syndrome.

Responders identified 3 broad areas of common interest. These included 1) medical updates, including a desire for access to detailed information about cancer risk and risk reduction strategies as well as assistance or advice on assembling a knowledgeable health care team, 2) shared experiences, expressing desire for an interactive forum for discussion of topics relevant and specific to people with Lynch syndrome and 3) psychosocial impact, including family dynamics, uncertainty, communication and general coping.

Thirty people attended the 2009 conference and forty-three attended the 2010 conference. Evaluations indicated a high level of satisfaction overall, with open-ended comments particularly in the areas of shared experiences and access to detailed, updated information. People requested additional conferences and longer meeting times. At least 3 individuals sought additional genetic counseling for themselves or family members after the conference.

## Conclusions

• Individuals and families with Lynch syndrome are seeking additional educational opportunities and psychosocial support resources post-genetic counseling.

• There is sufficient need and interest among this patient population to justify providing an organized venue for specific, detailed, updated information and psychosocial support.

• Evaluations indicate that the annual conferences were well-received and met the stated education and support goals.

